# Preparation and radiolabeling of a lyophilized (kit) formulation of DOTA-rituximab with ^90^Y and ^111^In for domestic radioimmunotherapy and radioscintigraphy of Non-Hodgkin’s Lymphoma

**DOI:** 10.1186/2008-2231-22-58

**Published:** 2014-07-29

**Authors:** Nazila Gholipour, Amir Reza Jalilian, Ali Khalaj, Fariba Johari-Daha, Kamal Yavari, Omid Sabzevari, Ali Reza Khanchi, Mehdi Akhlaghi

**Affiliations:** Department of Radiopharmacy, Faculty of Pharmacy, Tehran University of Medical Sciences, P.O. Box: 14155–6451, Tehran, Iran; Radiation Application Research School, Nuclear Science and Technology Research Institute, P.O. Box: 14395–836, Tehran, Iran; Department of Medicinal Chemistry, Faculty of Pharmacy, Tehran University of Medical Sciences, P.O. Box: 14155–6451, Tehran, Iran; Depatment of Toxicology and Pharmacology, School of Pharmacy, Tehran University of Medical Sciences, P.O. Box: 14176–14411, Tehran, Iran; Research Center for Nuclear Medicine, Tehran University of Medical Sciences, P.O. Box: 14117–13137, Tehran, Iran

**Keywords:** Rituximab, ^90^Y, ^111^In, Lymphoma-B, Biodistribution, Radioimmunotherapy

## Abstract

**Background:**

On the basis of results of our previous investigations on ^90^Y-DTPA-rituximab and in order to fulfil national demands to radioimmunoconjugates for radioscintigraphy and radioimmunotherapy of Non-Hodgkin’s Lymphoma (NHL), preparation and radiolabeling of a lyophilized formulation (kit) of DOTA-rituximab with ^111^In and ^90^Y was investigated.

**Methods:**

^111^In and ^90^Y with high radiochemical and radionuclide purity were prepared by ^112^Cd (p,2n)^111^In nuclear reaction and a locally developed ^90^Sr/^90^Y generator, respectively. DOTA-rituximab immunoconjugates were prepared by the reaction of solutions of p-SCN-Bz-DOTA and rituximab in carbonate buffer (pH = 9.5) and the number of DOTA per molecule of conjugates were determined by transchelation reaction between DOTA and arsenaso yttrium(III) complex. DOTA-rituximab immunoconjugates were labeled with ^111^In and ^90^Y and radioimmunoconjugates were checked for radiochemical purity by chromatography methods and for immunoreactivity by cell-binding assay using Raji cell line. The stability of radiolabeled conjugate with the approximate number of 7 DOTA molecules per one rituximab molecule which was prepared in moderate yield and showed moderate immunoreactivity, compared to two other prepared radioimmunoconjugates, was determined at different time intervals and against EDTA and human serum by chromatography methods and reducing SDS-polyacrylamide gel electrophoresis, respectively. The biodistribution of the selected radioimmunoconjugate in rats was determined by measurement of the radioactivity of different organs after sacrificing the animals by ether asphyxiation.

**Results:**

The radioimmunoconjugate with approximate DOTA/rituximab molar ratio of 7 showed stability after 24 h at room temperature, after 96 h at 4°C, as the lyophilized formulation after six months storage and against EDTA and human serum. This radioimmunoconjugate had a biodistribution profile similar to that of ^90^Y-ibritumomab, which is approved by FDA for radioimmunotherapy of NHL, and showed low brain and lung uptakes and low yttrium deposition into bone.

**Conclusion:**

Findings of this study suggest that further investigations may result in a lyophilized (kit) formulation of DOTA-rituximab which could be easily radiolabeled with ^90^Y and ^111^In in order to be used for radioimmunotherapy and radioscintigraphy of B-cell lymphoma in Iran.

## Introduction

Non-Hodgkin’s Lymphoma (NHL) also known as B and T cell lymphoma is a form of blood cancer originating in lymphatic system. Many investigations for treatment of NHL have been based on the development of antibody against CD-20 antigens which are expressed on the surface of B-cells [1]. Results of these investigations have led to drugs such as rituximab [2, 3], a chimeric antibody for immunotherapy of CD20-positive low-grade NHL, and ibritumomab and tositumomab derived from immortalized mouse cells for the treatment of follicular lymphoma [4]. Since NHL is multifocal and radiosensitive [1, 5, 6], anti-CD20 monoclonal antibodies (mAbs) labeled with ^111^In (^111^In-ibritumomab) for imaging, ^90^Y (^90^Y- ibritumomab, Zevalin) for therapy and ^131^I (^131^I-tositumomab, Bexxar) for imaging and therapy have been approved for use in patients with NHL [4, 7, 8]. It has been shown that the response rate of NHL in radioimmunotherapy (RIT) with ^90^Y-ibritumomab tiuxetan compared to rituximab immunotherapy is higher and unlike chemotherapy is not associated with severe mucositis, hair loss, or persistent nausea [1, 5, 8–10].

Radiolabeled Murine antibodies compared to those of humanized chimeric antibodies have shorter *in vivo* half-lives [1, 5] and as a result lower toxicities due to non-specific body irradiation. However these antibodies as foreign proteins may induce immunologic and anaphylactic reactions [11] in patients because of development of human anti murine antibody (HAMA) response and have not been approved for re-injection or retreatment [5, 7, 12]. Consequently, considerable investigations have been carried out for the preparation of radioimmunoconjugates from rituximab, a chimeric antibody which induce distinctly less antibody responses and preparation of rituximab labeled with ^90^Y [13, 14], ^111^In [15], ^153^Sm [16], ^177^Lu [17–19], ^64^Cu [20], ^149^ Tb, ^213^Bi, ^227^Th and ^225^Ac [11], through an additionally attached chelating agents such as different chemical forms of DTPA or DOTA [21, 22] onto antibody, have been described. Due to high costs of commercially available radiolabeled anti CD20 monoclonal antibodies and on the basis of results of our previous investigations on ^90^Y-DTPA-rituximab [14], where an unexpected high lung uptake was observed, and in the search for a radioimmunoconjugate of higher efficiency and lower toxicity, preparation of DOTA-rituximab which compared to DTPA-rituximab has higher stability appeared of interest. The aim of the present study was to develop a lyophilized formulation of DOTA-rituximab that could be labeled with ^111^In and ^90^Y in order to fulfil national demands to radioimmunoconjugates for radioscintigraphy and radioimmunotherapy of Non-Hodgkin’s Lymphoma (NHL). While preparation of ^90^Y-DOTA-rituximab has been reported [13], but characteristics, stability at different time intervals and different temperatures and biodistribution of the prepared radioimmunoconjugate have not been explained in details. This manuscript describes the synthesis, purification, and chemical characterization of a lyophilized (kit) DOTA-rituximab conjugate for labeling with ^90^Y and ^111^In and the radiochemical purity, in-vitro stability and biodistribution of the resulting radioimmunoconjugates.

## Materials and methods

### Materials

p-SCN-Bz-DOTA with %94 purity was obtained from Macrocyclics Inc. (NJ, USA). Rituximab (Reditux) was a pharmaceutical sample purchased from Cinnagen Co. (Tehran, Iran). Radio-TLC scanning was performed using a Bioscan AR-2000 radio TLC scanner instrument (Bioscan, Paris, France). A high purity germanium (HPGe) detector coupled with a Canberra™ (model GC1020-7500SL) multichannel analyzer and a dose calibrator ISOMED 1010 (Dresden, Germany) were used for counting radioactivity of organs of rats in biodistribution study. Analytical HPLC was performed by Shimadzu LC-10AT, armed with flow scintillation analyzer (Packard-150 TR) and UV-visible (Shimadzu) equipped with Whatman Partisphere C-18 column 250 × 4.6 mm, (Whatman Co. NJ, USA). Animal studies were performed in accordance with the United Kingdom Biological Council’s Guidelines on the Use of Living Animals in Scientific Investigations, 2nd Edn. which was approved by Animal Ethics Committee of the Deputy of Research of Nuclear Science and Technology Research Institute (NSTRI) of Iran.

### Preparation and quality control of ^111^In-InCl_3_

^111^In-InCl^3^ solution was produced by ^112^Cd (p,2n)^111^In nuclear reaction using a 30 MeV cyclotron in Agricultural, Medical and Industrial Research School (AMIRS) and purified by cation exchange chromatography on Dowex 50×8 resin. Gamma spectroscopy of the final sample was carried out by counting in an HPGe detector. Differential-pulsed anodic stripping polarography was used to ensure that the amount of cadmium, indium and copper ions in the sample are not higher than those of internationally accepted limits. The ^111^In-InCl_3_ solution was evaporated. The residue was dissolved in ultra-pure water and filtered under sterile condition. The radiochemical purity of the final ^111^In solution was checked in 10 mM DTPA (pH = 5.0) solution (R_f_ of free ^111^In^3+^ = 0.8) [15, 23].

### Preparation and quality control of ^90^Y-YCl_3_

^90^Y-YCl_3_ solution was prepared at Nuclear Science and Technology and Research Institute of Atomic Energy Organization of Iran by ion exchange chromatography technique through elution from an in-house made ^90^Sr/^90^Y generator which was developed for research purposes. The radionuclidic purity of the solution for the presence of other radionuclides was tested by beta spectroscopy using liquid scintillation counter (LSC). The radiochemical purity of ^90^YCl_3_ was checked by radio-TLC using 10 mM DTPA (pH = 5.0) solution (R_f_ of free ^90^Y ^3+^ = 0.8) [24].

### Preparation of DOTA-rituximab immunoconjugates

DOTA-rituximab immunoconjugates were prepared according to the reported method [18] with only slight modifications. Briefly, one milliliter of rituximab solution (10 mg/ml) was freed from excipients first by ultrafiltration using Vivaspin-2 filter (30 kDa, Sartorius AG, 2 × 10 min at 2.684 g) and then by washing three times with carbonate buffer (1 ml, 0.2 M Na_2_CO_3_, pH = 9.5). The antibody was then removed from the upper part of filter using 3.5 ml of carbonate buffer (0.2 M Na_2_CO_3_, pH = 9.5). The final concentration of rituximab was determined by a biophotometer (Eppendorf) at OD = 280 nm and found to be 2.73 mg/ml. The structural integrity of the antibody was confirmed by reducing SDS-PAGE electrophoresis [13]. 1.1 ml aliquots of resulting rituximab (3 mg, 2 × 10^-5^ mmol/1.1 ml) solution in three sterile and pyrigen-free vials were treated with the solution of p-SCN-Bz-DOTA in carbonate buffer (0.2 M, pH = 9.5) in molar ratios of 5 (0.073 mg, 10^-4^ mmol), 15(0.22 mg, 3 × 10^-4^ mmol), and 25 (0.36 mg, 5 × 10^-4^ mmol), respectively. The solutions were gently mixed for 20 times by pipetting up and down and then incubated at room temperature for 24 h. The coupling reactions were terminated and unreacted p-SCN-Bz-DOTA were removed by adjustment of the pH of solutions to 7.0 using ammonium acetate buffer (0.25 M, pH = 5.5), followed by ultrafiltration and then washing three times with ammonium acetate buffer (0.25 M, pH = 5.5). The immunoconjugates were then removed from upper part of filters with 1 ml aliquots of ammonium acetate buffer (0.25 M, pH = 5.5). The concentration of the antibody in final immunoconjugate solutions were measured by a biophotometer and found to be around 2.84 mg/ml. The antibody solutions were dispensed in sterile vials at a quantity of 0.3 mg per vials and treated with 30 μl aliquots of mannitol solution (mannitol Ph Eur, 80 mg/ml in ultra-pure water). Finally, the formulated immunoconjugate antibody solutions were freeze dried to obtain white pellets which were stored at -18°C.

### Determination of the number of DOTA molecules per one rituximab molecule in DOTA-rituximab immunoconjugates

The DOTA to rituximab ratios were determined on the basis of a transchelation between DOTA and arsenaso yttrium (III) complex from a standard curve for absorbance of arsenaso yttrium (III) complex at 652 nm which was constructed for a series of solutions containing 8.1 μM AAIII, 3.9 μM Y(III) and different concentrations of p-SCN-Bz-DOTA (0–20 μM) in 0.15 M ammonium acetate buffer (pH = 7) [25].

### Determination of the stability of the lyophilized (Kit) DOTA-rituximab immunoconjugates

Stability and degradation of the lyophilized DOTA-rituximab immunoconjugate kit (with selected DOTA:antibody ratio) was checked by integrity test using reducing SDS-polyacrylamide gel electrophoresis (reducing SDS-PAGE) at frequent monthly intervals (for 6 months) according to the method of Laemmli [26].

### Radiolabeling and quality control of the radiolabeled immunoconjugates

Typically, 510 MBq of ^111^InCl_3_ solution in 0.2 M HCl in conical vials were dried under a flow of nitrogen and residues were dissolved in 1500 μl of ammonium acetate buffer (pH = 5.5). 500 μl aliquots of ammonium acetate buffer were added to the vials containing lyophilized immunoconjugates (three different DOTA: antibody ratios) and the vials were mixed using pipetting up and down (10-20×) to dissolve immunoconjugates. Then 500 μl (170 MBq) aliquots of ^111^In solution were added to vials and after mixing gently for 5 min by up and down pipetting, mixtures were incubated at 37°C for 1–2 h.

The radiochemical purity of the products was determined by ITLC on Whatman No.2, using 10 mM DTPA as mobile phase and HPLC on a C-18 column using a gradient system as a reported routine method in this laboratory [27]. To increase the radiochemical purity, the radioimmunoconjugates were purified by chromatography using PD-10 columns (GE healthcare) and ammonium acetate buffer (pH = 7.0) as eluting solvent. The final solutions were then passed through 0.22 micron biological filters for stability and biodistribution studies. Radiolabeling of the immunoconjugates with ^90^Y and quality control of the resulting radioimmunoconjugate were carried out by the same method which was described for the preparation and quality control of ^111^In-DOTA-rituximab.

### Determination of the Immunoreactivity of radioimmunoconjugates

The immunoreactivity of radioimmunoconjugates were determined by the use of the cell-binding assay on Raji cell line, using 5 sequential dilutions of 10^6^-10^7^cells in exponential growth (microscopic determination generally indicated >90% viability). A known amount (6 ng) of antibody labeled with 2.54 MBq of ^111^In and ^90^Y, was incubated with cells in 200 μl of phosphate-buffered saline (0.15 molar NaCl and 0.01 molar phosphate buffer, pH = 7.0) containing 5% fetal calf serum and NaN_3_ (0.02% w/v) for 2 h at 37°C. Nonspecific binding, generally less than 3%, was assessed by competition with 100 μg of unlabeled antibody and results were used for determination of specific binding which were analyzed at maximal observed binding on 10^7^ cells by extrapolating to infinite antigen excess according to the reported method [28]. The Student T-test for paired samples was used for statistical comparison of results.

### Determination of the stability of radiolabeled DOTA-rituximab against EDTA

A 0.1 ml aliquot of the radiolabeled product with approximate number of 7 molecules of DOTA per rituximab molecule was mixed with 0.1 ml of ethylenediaminetetraacetic acid (30 mM, pH = 7.4) and allowed to incubate for 4 h at room temperature. The radiochemical purity of incubated solution was evaluated by radio-TLC [15].

### Determination of the stability of radiolabeled DOTA-rituximab in the presence of human serum

^111^In- and ^90^Y-DOTA-rituximab mixtures were incubated in freshly prepared human serum for 12 h at 37°C. Radiolabeling stability was assessed by size exclusion chromatography on a Sepharose column (1 × 30 cm) and using phosphate buffer solution as eluent at a flow rate of 0.5 ml/min. To obtain elution chromatogram, the incubated mixture was applied on the column and 1 ml fractions were collected and their radioactivities were determined. To calibrate the column, the control samples including free ^111^In and ^90^Y, radiolabeled human serum albumin and radiolabeled DOTA-rituximab were separately applied to the column for retention volume determination [15, 16].

### Radiolabeling efficiency of the lyophilized (Kit) DOTA-rituximab conjugates

The radiolabeling efficiency of the lyophilized DOTA-rituximab immunoconjugate (kit) was investigated by monthly radiolabeling with ^111^In and ^90^Y and ITLC analysis [15].

### Determination of the stability of the lyophilized (kit) radioimmunoconjugates

For evaluation of the stability of radiolabeled DOTA-rituximab, the radiochemical purities of samples of the final solution after storage at 4°C for 4 days and at 25°C for 24 h were determined by radio-TLC analysis [15]. Integrity of radiolabeled DOTA-rituximab was checked by reducing SDS-polyacrylamide gel electrophoresis [26].

### Determination of biodistribution of ^111^In-DOTA-rituximab and ^90^Y-DOTA-rituximab in rats

Male rates weighing 250-300 g were randomly divided into four groups. The first and second groups were administrated intravenously 0.3 mCi/kg aliquots of ^111^In-Rituximab and ^90^Y-rituximab solutions, respectively. The third and fourth groups were administered intravenously 250 mg/m^2^ of unlabeled rituximab, 2 hours before injection 0.3 mci/kg aliquots of ^111^In-Rituximab and ^90^Y-rituximab, to block CD-20 positive binding sites on the B-cells in the circulation and spleen [9]. The animals were sacrificed by ether asphyxiation at the exact time intervals, and the distribution of radioactivity, represented as percent of injected dose per gram (%ID/g), was determined by measurement of radioactivity of samples from different organs using a beta-scintillator detector for ^90^Y samples and an HPGe detector for counting the area under the curve of the 171 keV peak for ^111^In [15, 16].

## Results

### Preparation and quality control of radionuclides

^111^In as ^111^InCl_3_ solution with a specific activity of higher than1.55 GBq/μg indium at time of calibration was obtained by a non-carrier-added method. Quality control of the radionuclidic [15] showed the presence of 171 and 245 keV gamma energies, originating from ^111^In and a purity of higher than %99. The concentrations of cadmium (from target material) and copper (from target support) were 0.1 ppm which was below the internationally accepted levels and the radiochemical purity of ^111^In-InCl_3_ was higher than %98.

**Figure 1:**
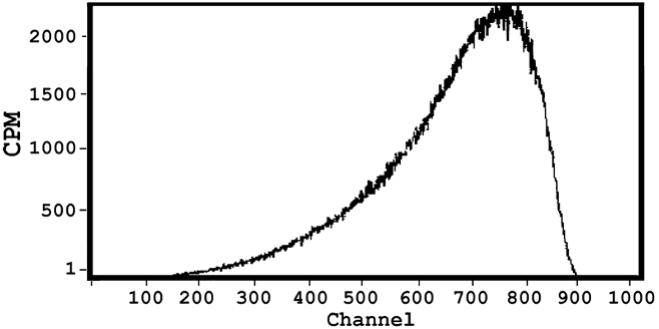
**Beta spectrum for** ^**90**^ **Y eluted from the** ^**90**^ **Sr/** ^**90**^ **Y generator sample.**

^90^Y was obtained from the natural decay of its parent, ^90^Sr (t_1/2_, 29 y) by an in house designed ^90^Sr/^90^Y generator which was developed for the research purpose. ^90^Sr was the only radionuclide impurity resulting from the production process and in all the yttrium (^90^Y) chloride solutions eluted from the generator the ^90^Sr/^90^Y ratio was ≤ 10^-5^. Figure 1 shows beta spectrum for Y-90 eluted from the ^90^Sr/^90^Y generator [24].

### Determination of the number of DOTA molecules per one rituximab molecule in DOTA-rituximab conjugates

**Figure 2:**
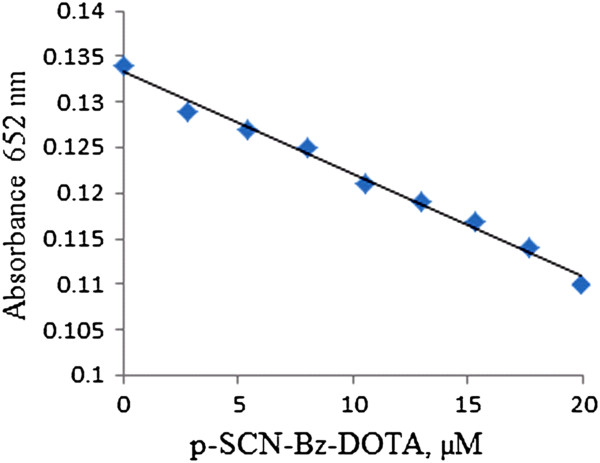
**The relationship between the absorbance of the complex and the molarities of p-SCN-Bz-DOTA at 652 nm.**

Figure 2 shows dependency of absorbance of arsenaso yttrium (III) complex at 652 nm on molarities of p-SCN-Bz-DOTA which was determined by arsenazo spectrophotometric method [25]. The solid line represents linear relationship between the absorbance, A_652nm_ and the concentration of 0–20 μM of p-SCN-Bz-DOTA. The approximate numbers of chelating group per rituximab molecule for immunoconjugates which were obtained by using different molar ratios of reactants are presented in Table 1.

**Table 1:** **The approximate number of DOTA molecules per rituximab molecule, radiolabeling yields, specific activity and radioimmunoreactivity of the prepared immunoconjugates and radioimmunoconjugates**

Rituximab: DOTA molar ratio	Approximate number of DOTA per rituximab	Radiolabeling yield for ^111^In (%) ^a^	Radiolabeling yield for ^90^Y (%) ^a^	Specific activity (MBq/mg) ^b^	
				^111^In	^90^Y
1:5	4	55.3	49.5	358	312
1:15	7	84	78	463	431
1:25	9	92	84	514	455

^a^Labeling conditions; 0.3 mg of immunoconjugate, 170 MBq of radioisotope, ammonium acetate buffer pH = 5.5, 37°C.

^b^Specific activity is calculated based on measured antibody concentration (UV) and radioactivity (dose calibrator).

### Radiolabeling and quality control of the immunoconjugates

**Figure 3:**
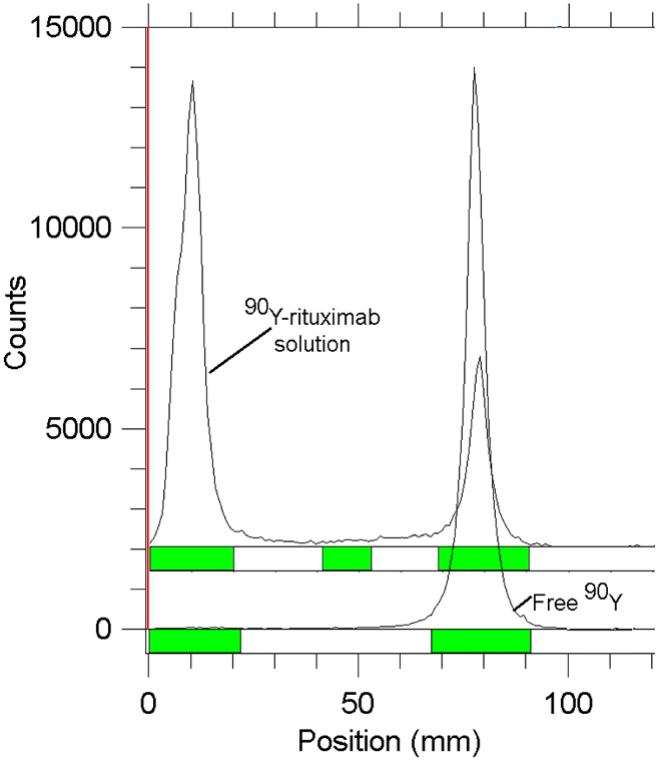
**Radio-TLC chromatograms of** ^**90**^ **YCl**_**3**_ **solution and** ^**90**^ **Y-DOTA-rituximab solution.**

The yields for radiolabeling of immunoconjugates are presented in Table 1. In radio-TLC experiments, the best eluent system for free ^90^Y and ^111^In detection was 10 mM DTPA aqueous solution (R_f_ of 0.8). Due to the size and charge of the protein (≈150,000 D), radiolabeled DOTA-rituximab remained at the sample origin but radiolabeled DOTA showed high R_f_ values. Figure 3 shows the radiochromatograms of free ^90^Y and ^90^Y-rituximab. Free ^111^In and ^111^In-rituximab pair showed similar radiochromatograms.

**Figure 4:**
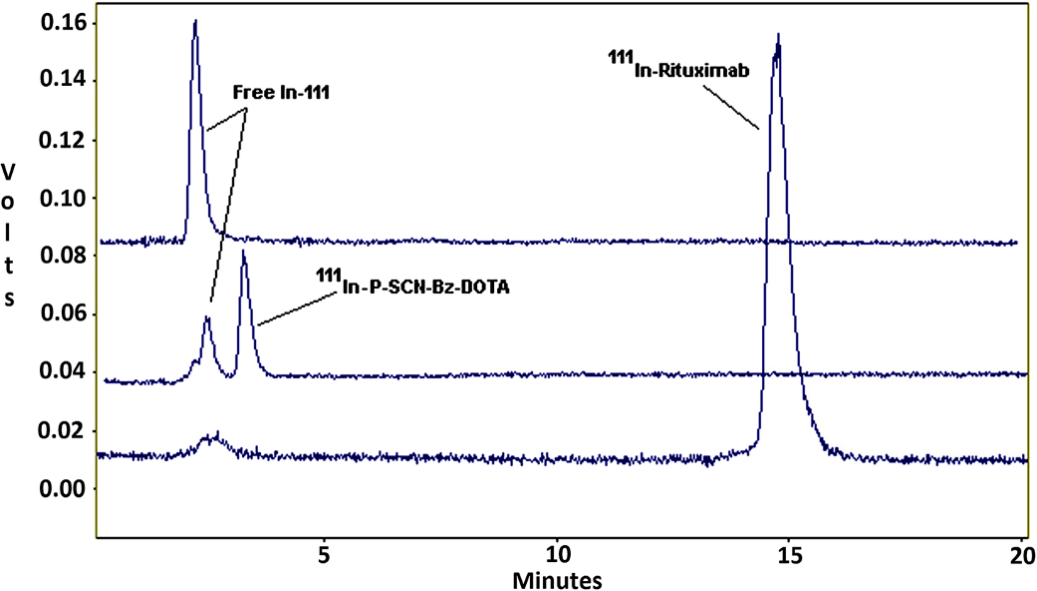
**HPLC chromatograms of free** ^**111**^ **In,** ^**111**^ **In-DOTA and** ^**111**^ **In-rituximab.**

Figure 4 shows HPLC chromatograms of free ^111^In, ^111^In-*P*-SCN-Bz-DOTA and ^111^In-rituximab. Both free indium and radiolabeled DOTA eluted rapidly but ^111^In-DOTA-rituximab was eluted at 14.8 min. ^90^Y, ^90^Y-*P*-SCN-Bz-DOTA and ^90^Y-DOTA-rituximab showed similar radiochromatograms.

### Results for the immunoreactivity of radioimmunoconjugates

The average immunoreactivity for radioimmunoconjugates with approximate DOTA/rituximab ratios of 4, 7 and 9 were %91.4, %72.8 and %47.3, respectively.

### Results for the stability of radioimmunoconjugate against EDTA and human serum

Table 2 represents radiochemical stability of radioimmunoconjugates which were checked by radio-TLC analysis at 4°C after 4 days and at 25°C after 24 h. There was a decrease of about 1-2% in radiochemical purity of radioimmunoconjugates in the presence of EDTA, indicating of their stabilities [15].

**Table 2:** **In vitro stability of** ^**111**^ **In-DOTA-rituximab**

Incubation time (h)	Radiochemical purity (%) ^a^	
	25°C	4°C
0	96.4 ± 1.7	96.4 ± 1.7
6	96.1 ± 2.1	96.3 ± 1.4
12	95.7 ± 2.3	94.5 ± 2.7
24	96.6 ± 1.2	95.9 ± 1.4
48	ND^b^	94.8 ± 2.6
72	ND	95.7 ± 1.5
96	ND	96.3 ± 1.3

^a^For each time point, n = 3.

^b^Not Determined.

^111^In- and ^90^Y-DOTA-rituximab were also stable in the presence of the fresh human serum. Analysis of aliquots of the samples after 24 h incubation by size exclusion chromatography showed that %96-98 of the radioactivity was associated with the radiolabeled DOTA-rituximab [13, 14].

**Figure 5:**
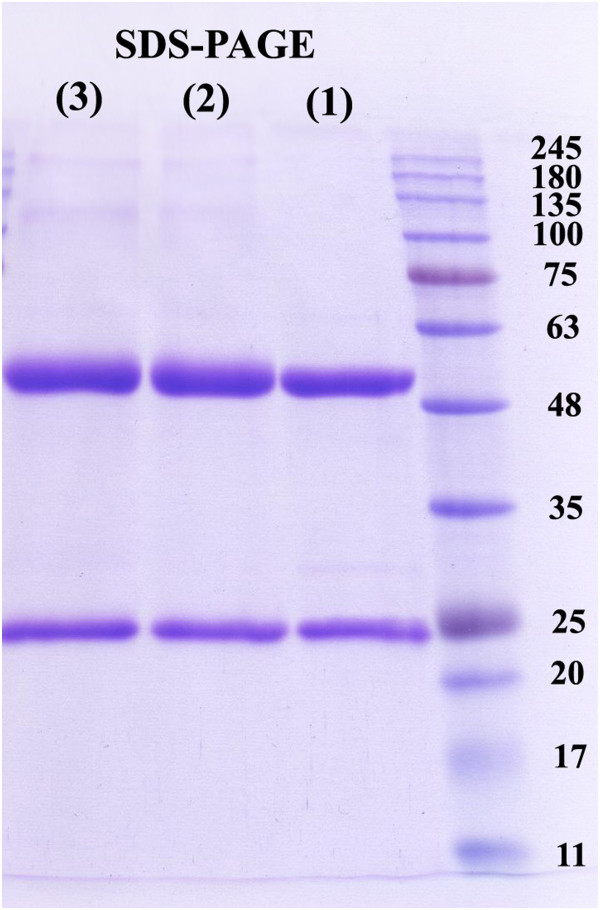
**Reducing SDS-PAGE lane patterns for rituximab (1), DOTA-rituximab conjugate (2) and** ^**90**^ **Y-DOTA-rituximab conjugate (3).**

As it is shown in Figure 5, the reducing SDS-PAGE patterns for rituximab, DOTA-rituximab and ^90^Y-DOTA-rituximab immunoconjugates were similar. Interestingly, the reducing SDS-PAGE results compared with a result for a commercially available rituximab sample showed no clear indication for antibody degradation. Similar results were obtained for monthly integrity tests on stored DOTA-rituximab immunoconjugate kits. The efficiencies of indium-111 radiolabeling of DOTA-rituximab kit (DOTA/antibody ratio = 7), measured monthly for six months, were 85.2 ± 3.1, 84.3 ± 1.7, 86.4 ± 2.8, 83.7 ± 3.4, 85.8 ± 1.9 and 84.8 ± 3.3, respectively. Reliable efficiencies also were observed for radioimmunoconjugate obtained by radiolabeling of DOTA-rituximab kit (DOTA/antibody ratio = 7) with ^90^yttrium.

### Results for the biodistribution study

**Figure 6:**
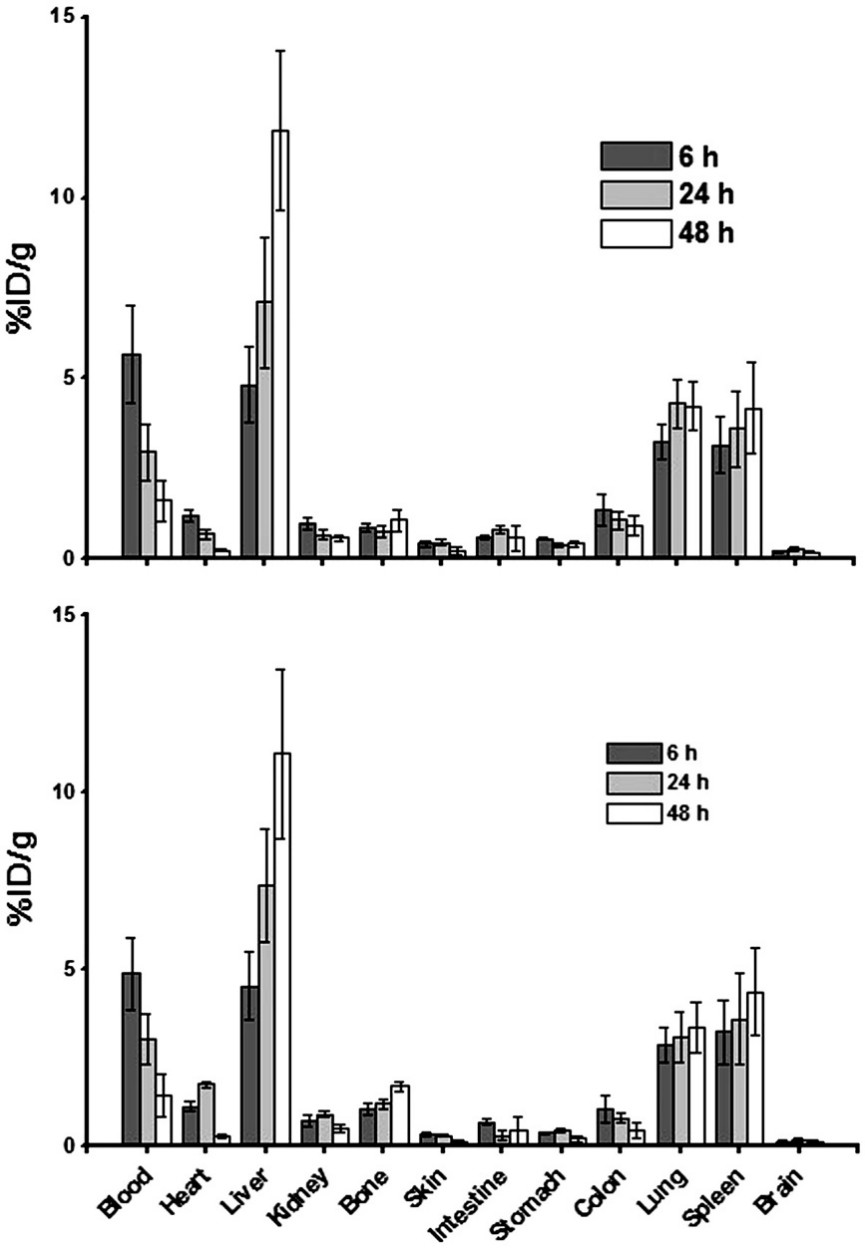
**Percentage of injected dose per gram (%ID/g) of** ^**111**^ **In-DOTA-Rituximab (up) and** ^**90**^ **Y-DOTA-rituximab (down) with approximate DOTA/rituximab molar ratio of 7 in rat organs.**

**Figure 7:**
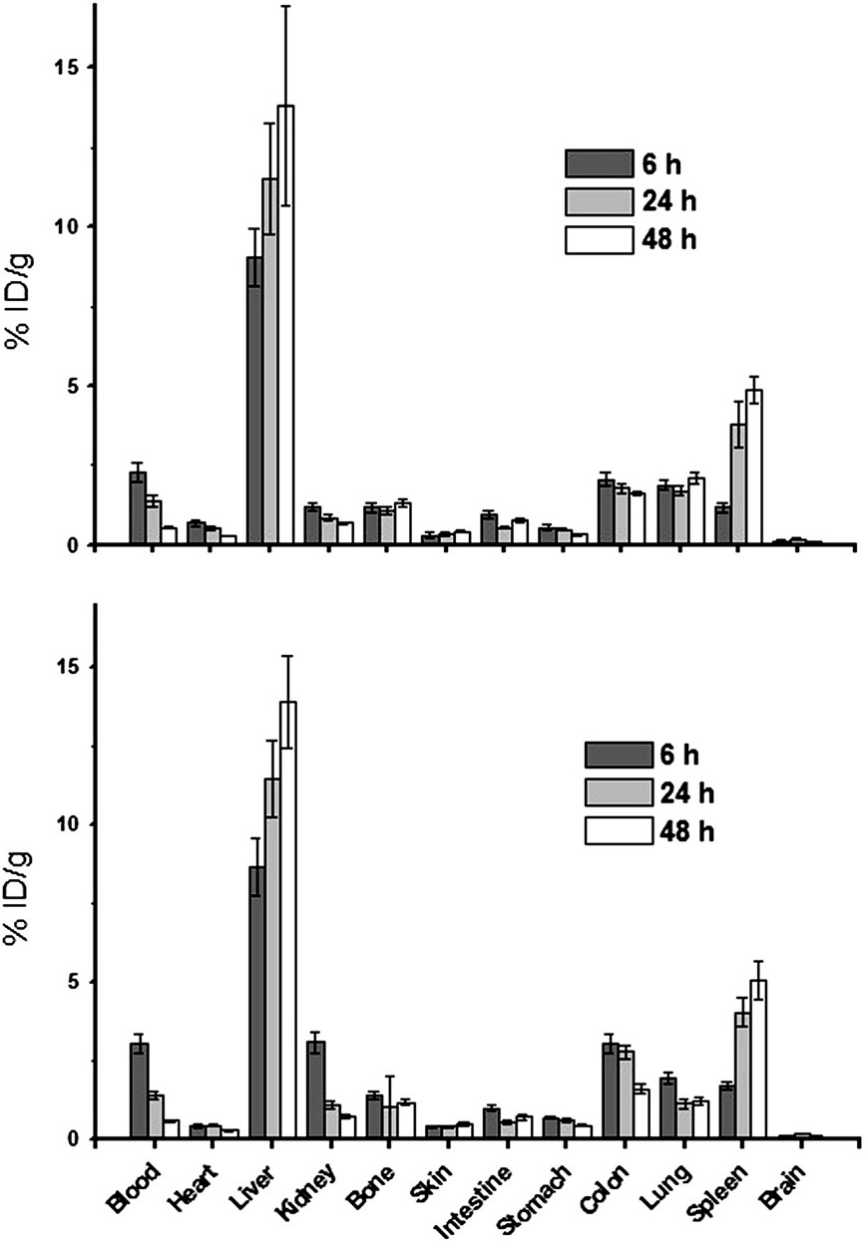
**Percentage of injected dose per gram (ID/g%) of** ^**90**^ **Y-DOTA-rituximab (up) and** ^**111**^ **In-DOTA-rituximab (down) with approximate DOTA/rituximab molar ratio of 7 in rat organs in rat organs, 250 mg/m** ^**2**^ **of unlabeled rituximab was injected 2 h before injection of radioimmunoconjugates.**

Figure 6 shows the biodistribution profiles of ^111^In-DOTA-rituximab and ^90^Y-DOTA-rituximab in male rats (first and second groups) which were determined by measurement of the radioactivities of the weighted samples of rat organs. Both groups showed similar biodistribution profiles. Over time, the liver and spleen uptakes were increased by reduction in the radioactivity of blood circulation. In the case of pre-injection of 250 mg/m^2^ of unlabeled rituximab (third and fourth groups), a faster clearance of radioactivity from blood circulation in comparison with first and second groups was observed. Figure 7 shows the biodistribution of ^111^In-DOTA-rituximab and ^90^Y-DOTA-rituximab after pre-injection of unlabeled rituximab. In these two groups, also biodistribution profiles were approximately similar. Lungs uptakes in groups 3 and 4 were lower compared to groups 1 and 2. Brain and bone showed very low uptake in all groups.

## Discussion

In this study preparation of a lyophilized (kit) formulation for radiolabeling of DOTA-rituximab with ^90^Y for radioimmunotherapy of B cells lymphoma and with ^111^In for radioimmunoscintigraphy as well as estimation of administered dose of ^90^Y-DOTA-rituximab was investigated. ^111^ In was prepared by ^112^Cd (p,2n)^111^In nuclear reaction and ^90^Y was prepared by^90^Sr/^90^Y generator which was developed locally for research purpose.

DOTA-rituximab immunoconjugates with approximate number of 4, 7 and 9 molecules of DOTA per one rituximab molecule were obtained by incubation of excess 5, 15 and 25 folds of p*-*SCN-benzyl-DOTA with rituximab using 0.2 M sodium carbonate buffer of pH = 9.5. DOTA-rituximab conjugates were successfully radiolabeled with ^111^In and ^90^Y, and the radiolabeling yields and efficiencies of radioimmunoconjugates which increased by increase in of DOTA/antibody molar ratios are reported in Table 1.

The immunoreactivity of radioimmunoconjugates compared to commercially available rituximab determined by Lindmo method [28] on Raji cells decreased by increase in DOTA/rituximab molar ratios and the average immunoreactivity for approximate molar ratios of 4, 7 and 9 were %91.4, %72.8 and %47.3, respectively. These findings suggest that increase in molar ratio of DOTA/antibody results to nonspecific conjugation of DOTA to antibody from antigen binding sites [29].

Due to the importance of both immunoreactivity and specific activity of radioimmunoconjugate and their oppositional behavior toward increasing the number of DOTA molecules per one antibody molecule, the immunoconjugate with approximate DOTA/antibody ratio of 7, with moderate immunoreactivity and specific activity, was selected for stability and bio-distribution studies. The selected radioimmunoconjugate showed stability when it was determined after 24 h at room temperature, after 96 h at 4°C, and after six months as lyophilized formulation. The selected radioimmunoconjugate was also stable to either EDTA or serum challenge for the duration of either experiment.

In agreement with results of studies on biodistribution of other labeled antibodies such as ^90^Y- ibritumomab, ^131^I-tositumomab and ^177^Lu-Anti CD-20 [9, 11, 17, 30], in this study after administration of ^111^In-DOTA-rituximab and ^90^Y-DOTA-rituximab with approximate DOTA/rituximab molar ratio of 7 in male rats the activity of blood circulation declined slowly and the activity of liver, spleen and other reticulloendothelial organs increased gradually. In the cases that rats were pre-treated with unlabeled rituximab the clearance of the radioactivity from the blood circulation was faster and the initial uptake of the activity in spleen was lower which could be attributed to blockade of CD-20 positive binding sites on B-cells in blood circulation and spleen [9]. The selected radioimmunoconjugate of this study also similar to ^90^Y-ibritumomab did not have any significant brain uptake and compared to ^90^Y-DTPA-rituximab of our previous investigation [14] showed significantly lower lung accumulation and ^90^Y deposition in the bone which may be explained by possible cross-linking or cyclization of antibody with cyclic DTPA dianhydride [31] and lower stability of DTPA conjugates compared with DOTA conjugates.

## Conclusion

Results of this study showed that radiolabeling of the lyophilized formulation (kit) of DOTA-rituximab having approximate DOTA/rituximab molar ratio of 7 with ^90^Y and ^90^In results in radioimmunoconjugates with moderate radioimmunoreactivity, and good stability and biodistribution profile. These findings suggest that further investigations may result in a kit formulation which could be easily radiolabeled with ^111^In and ^90^Y and to be used for radioimmunotherapy and radioscintigraphy of B cells lymphoma in Iran.

## Abbreviations

DOTA: 1,4,7,10-tetraazacyclododecane-1,4,7,10-tetraacetic acid
p-SCN-Bz-DOTA: p-benzyl isothiocyanate-1,4,7,10-tetraazacyclododecane-1,4,7,10-tetraacetic acid
NHL: Non-Hodgkin’s lymphoma
TLC: Thin layer chromatography
HPLC: High pressure liquid chromatography
SDS-PAGE: Sodium dodecyl sulfate-polyacrylamide gel electrophoresis.
